# A comparison of clinical phenotype and outcomes in anti- melanoma differentiation-associated protein 5 dermatomyositis and anti-synthetase syndrome in a multiethnic cohort in Singapore

**DOI:** 10.3389/fmed.2026.1969582

**Published:** 2026-10-12

**Authors:** Choon-Guan Chua, Zhaoxiang Fan, Hwee-Pin Phua, Wei-Yen Lim, Gin Tsen Chai, Mona Manghani

**Affiliations:** 1 Department of Rheumatology, Allergy and Immunology, Tan Tock Seng Hospital, Singapore, Singapore; 2 Department of Epidemiology and Preventive Medicine, Tan Tock Seng Hospital, Singapore, Singapore; 3 Department of Respiratory and Critical Care Medicine, Tan Tock Seng Hospital, Singapore, Singapore

**Keywords:** anti-synthetase syndrome, dermatomyositis, interstitial lung disease, prognosis, rapidly progressive interstitial lung disease (RP-ILD), MDA5

## Abstract

**Objective:**

This study aimed to compare the clinical characteristics and outcomes of anti-MDA5 dermatomyositis (MDA5-DM) and anti-synthetase syndrome (ASyS) in a multi-ethnic Southeast Asian cohort, and identify features that facilitate phenotypic differentiation and prognostic stratification.

**Methods:**

Chart review was performed on consecutive idiopathic inflammatory myopathies (IIM) patients managed at our centre from January 2015 to June 2021. Patients were classified using the 2017 EULAR/ACR IIM criteria, 2017 European Neuromuscular Centre criteria for immune-mediated necrotising myopathy (IMNM), and Connor's criteria for ASyS. Overlap myositis (OM) was defined as IIM in association with an underlying connective tissue disease or in the presence of a myositis-associated autoantibody. Rapidly progressive interstitial lung disease (RP-ILD) was defined as dyspnoea and/or hypoxaemia with worsening radiologic interstitial lung changes within 3 months of respiratory symptoms onset.

**Results:**

Among 119 IIM patients, dermatomyositis was the predominant subtype (57.1%), followed by ASyS (23.5%), OM (15.1%), and IMNM (4.2%). Anti-MDA5 antibodies were detected in 35.3% of dermatomyositis. MDA5-DM and ASyS exhibited comparable demographic characteristics. Anti-Jo1 (*n* = 14) was the commonest anti-tRNA synthetase antibody detected amongst ASyS patients. Compared with ASyS, MDA5-DM demonstrated significantly more prominent cutaneous manifestations—cutaneous ulcers, palmar papules, violaceous rash, Gottron's sign, inverse Gottron's sign, periungual erythema, and heliotrope rash (all *p* < 0.017); musculoskeletal features were similar between groups. Median peak creatine kinase levels were significantly higher in ASyS than MDA5-DM patients (median [IQR] 1317 [269−2713] vs. 227 [100−518] U/L; Hodges–Lehmann estimate = 773.5 U/L, 95% CI 150−1,788 U/L). ILD occurred in 42.0% of all IIM-subtypes; 78.9% of dermatomyositis-ILD cases were anti-MDA5 positive. RP-ILD was identified in 14 patients, of which 8 cases were anti- MDA5-DM and 4 ASyS. MDA5-DM/ASyS patients with RP-ILD had higher median peak AST levels (median [IQR] 103.5 [88−343] vs. 59 [44−119] U/L; Hodges–Lehmann estimate = 51 U/L, 95% CI 8−191 U/L) and markedly increased mortality compared with those without RP-ILD (75.0% vs. 12.5%; absolute risk difference 62.5%, 95% CI 35.7%–89.3%). The 1-year survival rates were 18.8% (95% CI 1.1%–53.5%) among MDA5-DM patients with RP-ILD, 75.0% (95% CI 12.8%–96.1%) among ASyS patients with RP-ILD and 97.4% (95% CI 83.2%–99.6%) among MDA5-DM or ASyS patients without RP-ILD. Survival differed significantly across these 3 groups (log rank *p* < 0.001).

**Conclusion:**

In our cohort, MDA5-DM and ASyS shared high burden of ILD but differed in cutaneous manifestations, risk of RP-ILD and survival, highlighting important diagnostic and prognostic distinctions warranting further investigation and informing clinical management.

## Introduction

Idiopathic inflammatory myopathies (IIM) comprise a heterogenous group of autoimmune diseases defined by distinct clinical and serological phenotypes. Among the IIM-subtypes, anti-melanoma differentiation-associated protein 5 (anti-MDA5) antibody dermatomyositis (MDA5-DM) and anti-synthetase syndrome (ASyS) share similar clinical features by having a strong association with interstitial lung disease and may present similarly with inflammatory arthritis, mechanic's hands, liited or absent clinically apparent myositis and a lower association with cancer compared to other IIM-subtypes (1–3).

ASyS was first defined as a distinct clinical entity in 1990 by the presence of anti-amino-acyl-tRNA synthetase (anti-ARS) antibodies together with ILD, myositis, non-erosive arthritis, Raynaud's phenomenon, mechanic's hands and fever (2, 4). Despite the relatively unique clinical phenotypes associated with anti-ARS antibodies the absence of formally accepted and externally validated classification criteria, heterogeneity between anti-ARS subtypes and variable analytical performance of commercially available antibody assays have hampered further research of this IIM-subtype (2, 5–8).

MDA5-DM frequently presents with clinically amyopathic or hypomyopathic disease and may be complicated by rapidly progressive interstitial lung disease (RP-ILD). While it accounts for less than 2% of dermatomyositis cases in White populations, it comprises over 20% in Asian populations (9–12). In East Asian cohorts it is frequently complicated by RP-ILD, often refractory to treatment and associated with a high 6-month mortality rate of 28%–66% (9, 13–18). In contrast, ILD in ASyS is frequently non-rapidly progressive, although acute and rapidly progressive presentations do occur (19–22).

The overlapping manifestations in both MDA5-DM and ASyS may make clinical distinction difficult initially and this is compounded by the lack of highly sensitive and specific commercially available myositis-specific autoantibody (MSA) detection tools (1, 2, 7, 8). There is currently a lack of comparative data between anti-MDA5 and ASyS phenotypes in a Southeast Asian multi-ethnic population as most published studies have examined either disease separately or compared ASyS with dermatomyositis as a broad category, potentially obscuring clinically relevant differences between antibody subtypes. In this retrospective study we compare the clinical phenotype and outcomes of patients with MDA5-DM and ASyS attending a tertiary centre in Singapore to determine which clinical features distinguish these two overlapping IIM phenotypes and to examine whether their pulmonary course and outcomes differ.

## Materials and methods

We conducted a retrospective observational cohort study at a tertiary referral centre in Singapore. Medical records of consecutive adult patients with IIM between 1 January 2015 to 30 June 2021 were reviewed. Chart reviewers were not blinded to patient outcomes. A sub-group of MDA5-DM from our IIM cohort was previously reported (14).

Patients were classified as having IIM based on clinical assessment supported by the 2017 EULAR/ACR classification criteria, immune-mediated necrotising myopathy (IMNM) using the 2017 European Neuromuscular Centre criteria, ASyS using Connor's classification criteria for ASyS, and overlap myositis (OM) as IIM in association with an underlying connective tissue disease or in the presence of a myositis-associated autoantibody (MAA) (5, 23, 24). IIM-subtype classification adjudication was applied retrospectively following individual chart review. We defined RP-ILD as dyspnoea and/or hypoxaemia with worsening radiologic interstitial lung changes within 3 months of respiratory symptoms onset (25). Cancer-associated myositis (CAM) was defined as the diagnosis of cancer in the period of 3 years before and after onset of IIM symptoms (3).

Clinical information on disease manifestations, laboratory data, imaging data, presence of malignancy, treatment and outcomes was obtained from medical chart review. The following parameters were collected: age of symptoms onset, age of diagnosis, sex, clinical features, presence of ILD and RP-ILD, laboratory data at the time of IIM diagnosis, history of malignancy (including type, timing of diagnosis in relation to IIM, staging, and pathological classification), treatment for both IIM and malignancy, clinical outcomes, and cause of death. All patients were tested for the presence of MSA/MAAs. Whole blood samples were drawn into EDTA tubes to enable separation of plasma by centrifugation, permitting autoantibody testing for determination of the presence of MSAs and MAAs using the *Euroline Autoimmune Inflammatory Myopathies 16 Antigen Profile* panel immunoblot assay. The panel included anti-MDA5 and anti-ARS antibodies, including anti-Jo-1, anti-PL-7, anti-PL-12 and anti-EJ together with other myositis specific and myositis-associated antibodies. Weak positive results and the presence of multiple myositis-specific antibodies were reviewed in the context of the clinical phenotype. No further confirmatory testing with ELISA or immunoprecipitation was performed. Radiological findings were extracted from original radiology reports.

This study is exploratory in nature and therefore no formal sample size calculation was performed. Cause of death was extracted from clinical notes where available.

Ethics approval was granted by the Domain Specific Review Board of the National Healthcare Group, Singapore (DSRB 2020/00011, DSRB 2024/00158). Waiver of patient consent was granted by the DSRB.

### Statistical analysis

Descriptive analyses were first performed to characterise demographic, clinical, laboratory, and outcome differences across three study groups (MDA5-DM, ASyS, and the remaining IIM cohort). Age at onset of IIM was summarised as mean (SD). Comparisons of age at onset were performed using Student's t-test for two-group analyses and linear regression for three-group analyses. Laboratory parameters were summarised as median (IQR) and compared using the Kruskal–Wallis test owing to their skewed distributions. Categorical variables were compared using Pearson's chi-square test or Fisher's exact test, as appropriate. For variables showing an overall difference across the three groups, *post-hoc* pairwise comparisons were conducted to identify the specific groups contributing to the observed differences.

For each participant, follow-up was calculated from the date of IIM diagnosis to death or administrative censoring on 31 December 2021, whichever occurred first. Participants who were alive at the end of follow-up were censored accordingly. As the vast majority of diagnosis dates were recorded only to the month and year, survival time from diagnosis to death was calculated in months rather than days to avoid introducing arbitrary assumptions regarding the exact date of diagnosis. For patients with diagnosis and death recorded within the same month, a follow-up time of 0.5 months was assigned to permit inclusion in the survival analysis. Kaplan–Meier (KM) methods were used to estimate overall survival among MDA5-DM and ASyS patients according to RP-ILD status: (1) MDA5-DM or ASyS without RP-ILD, (2) MDA5-DM with RP-ILD, (3) ASyS with RP-ILD**.** Survival curves were compared using the log-rank test. Where the overall log-rank test was statistically significant, *post-hoc* pairwise comparisons between the three RP-ILD subgroups were performed to identify specific subgroups that differed. Kaplan–Meier estimates of 6-month, 1-year and 3-year survival rates, with corresponding 95% confidence intervals (CIs), were obtained for each subgroup. Mortality incidence rates were also calculated and reported as deaths per 100 person-years of follow-up with 95% CIs.

Missing data were handled using available-case analysis. Analyses for each variable were based on all available observations, and no imputation of missing values was performed.

For selected pairwise comparisons of skewed continuous variables, Hodges–Lehmann estimates and corresponding 95% CIs were calculated. For selected binary outcomes of clinical interest, between-group differences were quantified using absolute risk differences and corresponding 95% CIs.

Statistical significance was defined as a two-sided *p* < 0.05. For *post-hoc* pairwise comparison arising from analyses involving three groups (MDA5-DM, ASyS, and the remaining IIM cohort; and the three RP-ILD survival subgroups), a Bonferroni-adjusted significance threshold of *p* < 0.017 (0.05/3) was applied to account for multiple testing. Statistical analyses were performed using StataNow version 19.5 (StataCorp LLC, College Station, TX, USA), with the exception of Hodges–Lehmann estimates and corresponding 95% CIs, which were calculated using R version 4.4.0 (R Foundation for Statistical Computing, Vienna, Austria).

## Results

### Demographic data

Of the 119 IIM patients, 68 (57.1%) had dermatomyositis, 28 (23.5%) ASyS, 18 (15.1%) OM and 5 (4.2%) IMNM. Amongst the dermatomyositis patients, 24/68 (35.3%) were positive for anti-MDA5 Ab. Comparing MDA5-DM and ASyS patients, both groups comprised mainly females (58.3% vs. 64.3%) and were predominantly Chinese (62.5% vs. 82.1%). There was no significant difference in mean age at symptom onset (50.8 vs. 55.8 years; *p* = 0.160) between both groups. Refer to Tables 1, 2 for comparison of characteristics between MDA5-DM, ASyS and remaining IIM-subtype patients.

**Table 1:** Demographic, cutaneous and musculoskeletal features of patients with anti-MDA5 dermatomyositis (MDA5-DM), anti-synthetase syndrome (ASyS) and other IIM-subtypes.

Characteristics	MDA5-DM (*n* = 24)	ASyS (*n* = 28)	Other IIM-subtypes (*n* = 67)	Overall (*n* = 119)	Missing (*n* = 119)	Overall *p*-value	MDA5-DM vs. ASyS *p*-value
Demographics							
Age at onset							
Mean (standard deviation)	50.8 (11.8)	55.8 (13.5)	55.9 (15.9)	54.8 (14.7)	0 (%)	0.312	0.160
(minimum, maximum)	(29, 69)	(20, 84)	(20, 96)	(20, 96)			
Sex							
Female	14 (58.3%)	18 (64.3%)	48 (71.6%)	80 (67.2%)	0 (%)	0.457	0.66
Male	10 (41.7%)	10 (35.7%)	19 (28.4%)	39 (32.8%)			
Ethnicity							
Chinese	15 (62.5%)	23 (82.1%)	56 (83.6%)	94 (79.0%)	0 (%)	0.084	0.042
Malays	8 (33.3%)	3 (10.7%)	7 (10.4%)	18 (15.1%)			
Indians	0 (0.0%)	2 (7.1%)	3 (4.5%)	5 (4.2%)			
Others	1 (4.2%)	0 (0.0%)	1 (1.5%)	2 (1.7%)			
Clinical characteristics							
Cutaneous features							
Cutaneous ulcers	17 (70.8%)	1 (3.6%)	2 (3.0%)	20 (16.8%)	0 (%)	<0.001	<0.001
Palmar papules	8 (33.3%)	0 (0.0%)	0 (0.0%)	8 (6.7%)	0 (%)	<0.001	<0.001
Violaceous rash	15 (62.5%)	3 (10.7%)	9 (13.4%)	27 (22.7%)	0 (%)	<0.001	<0.001
Gottron's sign	14 (58.3%)	5 (17.9%)	35 (52.2%)	54 (45.4%)	0 (%)	0.003	0.003
Inverse gottron's	7 (29.2%)	0 (0.0%)	1 (1.5%)	8 (6.7%)	0 (%)	<0.001	0.003
Periungual erythema	12 (50.0%)	4 (14.3%)	32 (47.8%)	48 (40.3%)	0 (%)	0.004	0.007
Heliotrope rash	11 (45.8%)	4 (14.3%)	31 (46.3%)	46 (38.7%)	0 (%)	0.008	0.016
Mechanics hands	11 (45.8%)	10 (35.7%)	7 (10.4%)	28 (23.5%)	0 (%)	<0.001	0.458
Musculoskeletal features							
Proximal myopathy	14 (58.3%)	19 (67.9%)	54 (80.6%)	87 (73.1%)	0 (%)	0.083	0.477
Amyopathic	10 (41.7%)	7 (25.0%)	13 (19.4%)	30 (25.2%)	0 (%)	0.098	0.202
Polyarthritis	13 (54.2%)	8 (28.6%)	2 (3.0%)	23 (19.3%)	0 (%)	<0.001	0.061

**Table 2:** Lung involvement, laboratory results and other characteristics of patients with anti-MDA5 dermatomyositis (MDA5-DM), anti-synthetase syndrome (ASyS) and other IIM-subtype.

Characteristics	MDA5-DM (*n* = 24)	ASyS (*n* = 28)	Other IIM-subtypes (*n* = 67)	Overall (*n* = 119)	Missing (*n* = 119)	Overall *p*-value	MDA5-DM vs. ASyS *p*-value
Lung involvement							
Interstitial lung disease	15 (62.5%)	23 (82.1%)	12 (17.9%)	50 (42.0%)	0 (%)	<0.001	0.111
RP-ILD	8 (33.3%)	4 (14.3%)	2 (3.0%)	14 (11.8%)	0 (%)	<0.001	0.186
Laboratory results							
Median peak CK (IQR)	227 (100, 518)	1317 (269, 2713)	903.5 (188, 4487)	619 (169, 2419.5)	31 (26.1%)	<0.001	0.003
Median peak ALT (IQR)	86 (61.5, 195.5)	67.5 (39.5, 161)	99 (58, 223)	82 (49, 195)	34 (28.6%)	0.283	0.158
Median peak AST (IQR)	88.5 (54, 143)	59.0 (41, 123)	101 (70, 195)	84.5 (49, 151)	35 (29.4%)	0.293	0.282
Others							
Presence of cancer (within 3 years before and after IIM diagnosis)	2 (8.3%)	1 (3.6%)	22 (32.8%)	25 (21.0%)	0 (%)	<0.001	0.590
Death	8 (33.3%)	6 (21.4%)	15 (22.4%)	29 (24.4%)	0 (%)	0.517	0.335

Laboratory reference range: ALT 5–40 U/L; 15–40 U/L AST; CK 50–350 U/L. ALT, alanine transaminase; AST, aspartate transaminase; CK, creatine kinase; IIM, idiopathic inflammatory myopathies; IQR, inter-quartile range; RP-ILD, rapidly-progressive interstitial lung disease.

### Autoantibodies profile

Anti-ARS antibodies detected amongst the ASyS patients were anti-Jo1 (14/28, 50.0%), anti-PL7 (10/28, 35.7%), anti-PL12 (4/28, 14.3%), and anti-EJ (2/28, 7.1%). 8 ASyS had >1 MSA or MAAs (other than anti-Ro52) detected. Anti-Ro52 was detected in 14 (58.3%) MDA5-DM and 18/28 (64.3%) ASyS patients. Other MSAs detected in the 119 IIM patients were as follows: 39 anti-TIF1ɣ, 8 anti-SRP, 8 anti-Mi2beta, 4 anti-Mi2alpha, 4 anti-NXP2 and 2 anti-SAE1.

### Clinical characteristics

In terms of cutaneous manifestations, MDA5-DM had prominent cutaneous features compared to ASyS, particularly cutaneous ulcers, palmar papules, and violaceous rash (all *p* < 0.001). MDA5-DM also had more Gottron's sign (*p* = 0.003), inverse Gottron's (*p* = 0.003), periungual erythema (*p* = 0.007) and heliotrope rash (*p* = 0.016). No significant difference in presence of mechanics hands was observed in either IIM phenotype (MDA5-DM 45.8% vs. ASyS 35.7%, *p* = 0.458).

In regard to musculoskeletal manifestations, 19/28 (67.9%) ASyS and 14/24 (58.3%) MDA5-DM had proximal myopathy respectively (*p* = 0.477). 8/28 (28.6%) ASyS and 13/24 (54.2%) MDA5-DM had polyarthritis (*p* = 0.061). Polyarthritis trended higher in MDA5-DM but did not meet statistical significance.

Peak creatine kinase (CK) levels were significantly higher in ASyS than MDA5-DM patients (median [IQR] 1317 [269−2713] vs. 227 [100−518] U/L; Hodges–Lehmann estimate = 773.5 U/L, 95% CI 150−1,788 U/L). No significant differences were observed between ASyS and MDA5-DM patients in median peak ALT levels (Hodges–Lehmann estimate = −21 U/L, 95% CI −56 to 8 U/L) or AST levels (Hodges–Lehmann estimate = −14 U/L, 95% CI −46 to 10 U/L).

In the entire IIM cohort, 50/119 (42.0%) had ILD. Amongst the IIM-ILD, 23/50 (46.0%) had ASyS, 19/50 (38.0%) dermatomyositis, 7/50 (14.0%) OM and 1/50 (2.0%) IMNM. 15/19 (78.9%) dermatomyositis with ILD were positive for anti-MDA5 Ab. 14 patients had RP-ILD—8 dermatomyositis, 4 ASyS and 2 OM. Diagnosis of all RP-ILD was synchronous with diagnosis of IIM. All dermatomyositis RP-ILD patients were positive for anti-MDA5. Of the 4 ASyS RP-ILD, 3 were positive for anti-Jo1 and 1 anti-PL12. Of the 2 OM RP-ILD patients, 1 was positive for both anti-TIF1ɣ and anti-Scl75, and the other patient was positive for anti-Jo1 and anti-Ro52 with clinical features of scleroderma. Anti-Ro52 was present in 8/14 (57.1%) RP-ILD patients—4 MDA5-DM, 3 ASyS and 1 OM patients.

2/24 MDA5-DM and 3/28 ASyS patients had a history of cancer. Of these patients with cancer, 3 (2 MDA5-DM and 1 ASyS) occurred within 3 years before and 3 years after the onset of IIM symptoms).

### MDA5-DM and ASyS with RP-ILD vs. without RP-ILD

Comparing all MDA5-DM and ASyS patients (MDA5-DM/ASyS) with RP-ILD against those without RP-ILD, no significant differences in cutaneous or musculoskeletal manifestation was observed. Higher median peak AST level was observed in MDA5-DM/ASyS with RP-ILD than those without RP-ILD (median [IQR] 103.5 [88−343] vs. 59 [44−119] U/L; Hodges–Lehmann estimate = 51 U/L, 95% CI 8−191 U/L). No significant differences in median peak CK level (Hodges–Lehmann estimate = −140.7 U/L, 95% CI −839 to 108 U/L) or ALT levels (Hodges–Lehmann estimate = 21.7 U/L, 95% CI −23 to 157 U/L) were observed between these two groups. Mortality amongst those with RP-ILD was significantly higher compared with those without RP-ILD (75% vs. 12.5%, absolute risk difference 62.5%, 95% CI 35.7%–89.3%).

No cancer was reported in the RP-ILD subgroup compared with the 5/40 among the non-RP-ILD group. See Table 3 for comparison of clinical characteristics between MDA5-DM and ASyS patients with and without RP-ILD.

**Table 3:** Comparison of clinical characteristics between anti-MDA5 dermatomyositis (MDA5-DM) and anti-synthetase syndrome (ASyS) patients with and without rapidly-progressive interstitial lung disease (RP-ILD).

Characteristics	With RP-ILD (*n* = 12)	Without RP-ILD (*n* = 40)	*p*-value
Demographics			
Age at onset			
Mean (standard deviation)	59.6 (10.2)	51.6 (13.1)	0.059
(minimum, maximum)	(41, 84)	(20, 76)	
Gender			
Female	7 (58.3%)	25 (62.5%)	0.795
Male	5 (41.7%)	15 (37.5%)	
Ethnicity			
Chinese	9 (75.0%)	29 (72.5%)	0.386
Malays	2 (16.7%)	9 (22.5%)	
Indians	0	2 (5.0%)	
Others	1 (8.3%)	0	
Clinical characteristics			
Cutaneous features			
Cutaneous ulcers	6 (50.0%)	12 (30.0%)	0.202
Palmar papules	3 (25.0%)	5 (12.5%)	0.366
Violaceous rash	5 (41.7%)	13 (32.5%)	0.558
Gottron's sign	4 (33.3%)	15 (37.5%)	1.000
Inverse Gottron's	3 (25.0%)	4 (10.0%)	0.331
Periungual erythema	6 (50.0%)	10 (25.0%)	0.100
Heliotrope rash	4 (33.3%)	11 (27.5%)	0.726
Mechanics hands	6 (50.0%)	15 (37.5%)	0.439
Musculoskeletal features			
Proximal myopathy	7 (58.3%)	26 (65.0%)	0.674
Amyopathic	5 (41.7%)	12 (30.0%)	0.450
Polyarthritis	6 (50.0%)	15 (37.5%)	0.439
Lung involvement			
Interstitial lung disease	12 (100%)	26 (65.0%)	0.004
Laboratory results			
Median peak CK (IQR)	320 (113, 505)	550.5 (166.5, 1829)	0.282
Median peak ALT (IQR)	120.5 (47.5, 308.5)	79 (42.5, 141)	0.409
Median peak AST (IQR)	103.5 (88, 343)	59 (44, 119)	0.025
Others			
Presence of cancer (within 3 years before and after IIM diagnosis)	0	5 (12.5%)	0.198
Death	9 (75.0%)	5 (12.5%)	<0.001

Laboratory reference range: ALT 5–40 U/L; 15–40 U/L AST; CK 50–350 U/L. Peak AST value was missing for one subject. ALT, alanine transaminase; AST, aspartate transaminase; CK, creatine kinase; IIM, idiopathic inflammatory myopathies; IQR, inter-quartile range; RP-ILD, rapidly-progressive interstitial lung disease.

### Treatment and prognosis

Glucocorticoids were the most commonly prescribed treatment to all MDA5-DM (100%) and ASyS (96.4%) patients. Intravenous pulsed methylprednisolone (IV MEP) was given to 14/24 MDA5-DM and 4/28 ASyS patients. The most common steroid-sparer prescribed for MDA5-DM were IV rituximab (*n* = 13) and calcineurin inhibitor (9 ciclosporin A, 4 tacrolimus) followed by tofacitinib (*n* = 10) and cyclophosphamide (*n* = 10). For ASyS, the most common steroid-sparer prescribed was azathioprine (*n* = 19) followed by cyclophosphamide (*n* = 10), calcineurin inhibitor (7 ciclosporin A, 1 tacrolimus), methotrexate (*n* = 9) and mycophenolate (*n* = 6).

For the RP-ILD patients, all 8 MDA5-DM and 4 ASyS patients received glucocorticoids. Of these, 7/8 MDA5-DM and 3/4 ASyS received IV MEP. IV cyclophosphamide was the most common steroid-sparer given (3/8 MDA5-DM, 4/4 ASyS) followed by IV rituximab (5/8 MDA5-DM, 0 ASyS) and oral ciclosporin A (4/8 MDA5-DM, 1 ASyS). 4 RP-ILD patients (all MDA5-DM) were given tofacitinib, of which 3 received MEP-RTX-TOF treatment.

Table 4 summarises the treatment prescribed for MDA5-DM and ASyS patients.

**Table 4:** Comparison of treatment prescribed for anti-MDA5 dermatomyositis (MDA5-DM), anti-synthetase syndrome (ASyS) patients.

Treatment	MDA5-DM (*n* = 24)	ASyS (*n* = 28)
IV methylprednisolone	14 (58.3%)	4 (14.3%)
Glucocorticoids (excluding IV methylprednisolone)	24 (100%)	27 (96.4%)
IV rituximab	13 (54.2%)	2 (7.1%)
Oral mycophenolate mofetil and mycophenolate sodium	2 (8.3%)	6 (21.4%)
Oral azathioprine	8 (33.3%)	19 (67.9%)
Oral ciclosporin A	9 (37.5%)	7 (25%)
Oral tacrolimus	4 (16.7%)	1 (3.6%)
Oral methotrexate	4 (16.7%)	9 (32.1%)
Oral tofacitinib	10 (41.7%)	0 (0%)
Intravenous immunoglobulin	6 (25%)	2 (7.1%)
Cyclophosphamide (IV or oral)	10 (41.7%)	10 (35.7%)

Treatment data are presented descriptively. No formal statistical comparisons were performed between groups.

### Survival analyses

A total of 52 patients were included in the survival analysis, contributing 3,730.5 person-months (310.9 person-years) of follow-up. One patient with diagnosis and death recorded within the same month was assigned a follow-up time of 0.5 months. The median follow-up duration was 62 months. (range: 0.5−390 months). During follow-up, 14 deaths were observed.

Kaplan–Meier survival curves stratified by subgroup are shown in Figure 1. Time zero for survival analysis was taken at the time of IIM diagnosis. The overall log-rank test showed a statistically significant difference in survival among the three subgroups (*p* < 0.001). *Post-hoc* pairwise comparisons showed no statistically significant difference in survival between MDA5-DM with RP-ILD patients and ASyS with RP-ILD patients (*p* = 0.186). The one-year survival rates of MDA5-DM with RP-ILD patients and ASyS with RP-ILD patients were 18.8% (95% CI: 1.1%–53.5%) and 75.0% (12.8%–96.1%). The 3-year survival rates of both groups remained the same. Given the relatively small sample size in subgroups with RP-ILD, these analyses should be interpreted as exploratory. Mortality incidence rates with corresponding 95% confidence intervals in addition to survival outcomes by subgroup are presented in Tables 5, 6.

**Figure 1:**
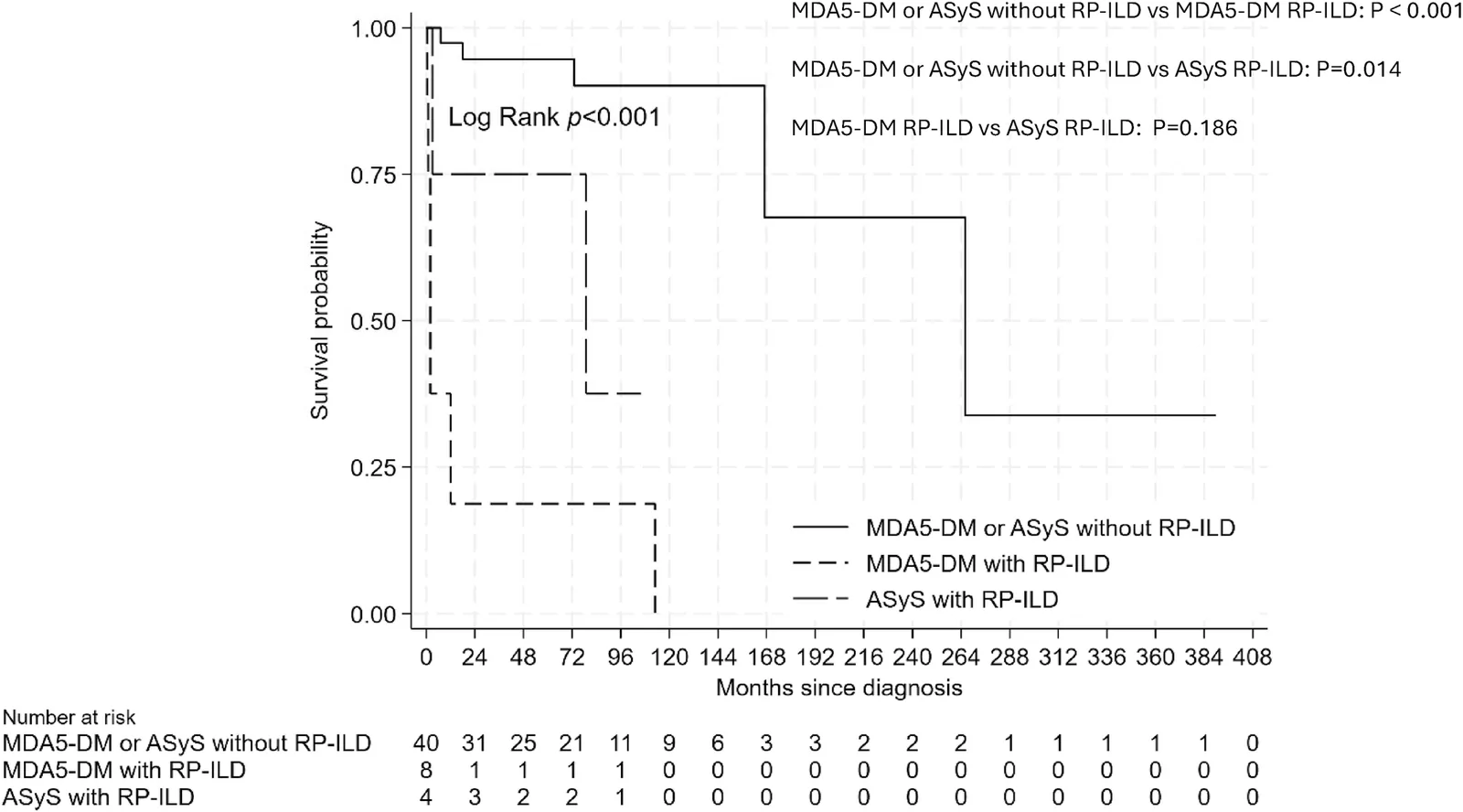
Kaplan–meier estimates of overall survival among (1) MDA5-DM or ASyS without RP-ILD, (2) MDA5-DM with RP-ILD, and (3) ASyS with RP-ILD. The table below the graph shows the number of patients remaining at risk at each time point. Survival curves were compared using the log-rank test.

**Table 5:** Mortality incidence rates among anti-MDA5 dermatomyositis (MDA5-DM) and anti-synthetase syndrome (ASyS) patients with and without rapidly-progressive interstitial lung disease (RP-ILD).

Subgroup	Number of subjects	Deaths	Time at risk (Person-years)	Mortality incidence rate per 100 person years (95% CI)
MDA5-DM or ASyS without RP-ILD	40	5	279.6	1.8 (0.7–4.3)
MDA5-DM with RP-ILD	8	7	12.0	58.5 (27.9–120.0)
ASyS with RP-ILD	4	2	19.3	10.3 (2.6–41.4)
Overall MDA5-DM/ASyS	52	14	310.9	4.5 (2.7–7.6)

**Table 6:** 6-, 12-months survival estimates among anti-MDA5 dermatomyositis (MDA5-DM) and anti-synthetase syndrome (ASyS) patients with and without rapidly-progressive interstitial lung disease (RP-ILD).

	6-month		1-year		
Subgroup	Survival Rate (%) (95% CI)	Deaths within period	Survival Rate (%) (95% CI)	Deaths within period	Median Survival Time (months)
MDA5-DM or ASyS without RP-ILD	100	0	97.4 (83.2–99.6)	1	266
MDA5-DM with RP-ILD	37.5 (8.7–67.4)	5	18.8 (1.1–53.5)	6	2
ASyS with RP-ILD	75.0 (12.8–96.1)	1	75.0 (12.8–96.1)	1	79
Overall MDA5-DM/ASyS	88.5 (76.1–94.6)	6	84.4 (71.3–91.9)	8	266

## Discussion

Our study focused on 2 IIM-subtypes (MDA5-DM and ASyS) which are known to have similar clinical features. We made general comparisons with other IIM-subtypes before identifying distinct differences between these 2 clinical phenotypes. 15/24 (62.5%) of MDA5-DM and 23/28 (82.1%) of ASyS patients had ILD as compared to 12/67 (17.9%) in the remaining IIM-subtypes (others) (all *p* < 0.001). Other distinguishing features were relatively higher prevalence of arthritis in both MDA5-DM and ASyS than in the remaining subtypes (54.2% vs. 3.0% and 28.6% vs. 3.0% respectively; both *p* < 0.001) and lower incidence of CAM in MDA5-DM and ASyS, although statistical significance was observed only for ASyS when compared with the remaining IIM subtypes (3.6% vs. 32.8%, *p* < 0.001).

The significant distinguishing features between MDA5-DM and ASyS patients identified in our study were (a) MDA5-DM had distinct cutaneous manifestations with higher frequencies of cutaneous ulcers, palmar papules, violaceous rash, Gottron’s sign, inverse Gottron’s, periungual erythema and heliotrope rash, (b) higher frequencies of RP-ILD in MDA5-DM, and (c) poorer prognosis of MDA5-DM RP-ILD compared with ASyS RP-ILD.

First, our study demonstrated that 15/24 (62.5%) of our MDA5-DM and 23/28 (82.1%) of ASyS subtype groups had significantly higher ILD prevalence as compared to 12/67 (17.9%) amongst the remaining IIM-subtypes group (both *p* < 0.001). The ILD prevalences in MDA5-DM and amongst ASyS in our cohort were similar to other patient registries and cohorts (26–29). The prevalence of RP-ILD was higher among patients with MDA5-DM than ASyS (absolute difference = 19.0%, 95% CI −3.9% to 42.0%) and markedly higher than among other IIM subtypes (absolute difference = 30.3%, 95% CI 11.0% to 49.7%). ASyS patients also exhibited a higher prevalence of RP-ILD than other IIM-subtypes (absolute difference = 11.3%, 95% CI −2.3% to 24.9%). Despite the lack of statistical significance, the absolute differences and corresponding CIs on RP-ILD prevalence between sub-groups do indicate that RP-ILD is more prevalent in MDA5-DM compared to ASyS and other IIM-subtypes. Our findings align with similar observation of higher prevalence of RP-ILD in MDA5-DM compared to ASyS reported by other authors (19, 29, 30).

Second, our study demonstrated a lower survival rate amongst MDA5-DM patients compared to ASyS. MDA5-DM RP-ILD patients had significantly lower survival rates than in ASyS RP-ILD and all other MDA5-DM/ASyS patients with no RP-ILD (*p* < 0.001). The 6-month survival rates for MDA5-DM RP-ILD and ASyS RP-ILD were 37.5% and 75.0% respectively. The corresponding 1-year survival rates were 18.8% and 75.0%, with no further deaths observed beyond the first year, resulting in unchanged survival rates at 3 years. Although based on a small number of patients and events, these findings suggest that MDA5-DM RP-ILD patients had the highest mortality rate within the first year after disease onset. In a direct comparison between 110 MDA5-DM RP-ILD and 70 ASyS RP-ILD patients, *Zuo* et al. had a similar observation of higher mortality rate in MDA5-DM RP-ILD patients within the first year of disease (19). Thus, despite the small sample size in our cohort, these findings were similar to those observed in other reports and studies.

Third, our study demonstrated specific DM-cutaneous features that could distinguish MDA5-DM from ASyS patients. These were namely presence of cutaneous ulcers, palmar papules, violaceous rash, Gottron's sign, inverse Gottron's, periungual erythema and heliotrope rash. Importantly, the presence of mechanics hands could not distinguish between MDA5-DM and ASyS. Significant differences in the presence of heliotrope rash, Gottron sign, V sign, shawl sign and skin ulcer were previously described when MDA5-DM and ASyS patients with RP-ILD were compared (19). However, further subgroup analyses to distinguish the clinical features between MDA5-DM/ASyS with RP-ILD compared to those without ILD was unyielding in our cohort.

Both MDA5-DM and ASyS had a significantly higher prevalence of arthritis compared with the remaining IIM subtypes. Although the prevalence of CAM was lower in both MDA5-DM and ASyS, the difference was statistically significant only for ASyS. The higher prevalence of arthritis and lower cancer prevalence for both MDA5-DM and ASyS were similarly observed in other reports and studies (1, 3, 20, 27, 28). However, the prevalence of arthritis observed in our ASyS (28.6%) appeared to be lower than the estimated 60−70% reported in other studies (27, 31, 32). We also observed that 58.3% of anti-MDA5 patients had proximal myopathy despite significantly lower median peak CK levels of 227 U/L compared to 1,317 U/L in ASyS (67.9% had proximal myopathy). This finding of muscle enzyme-proximal myopathy phenotype dissociation in MDA5-DM patients appear unique and has also been reported by Sama S et al. (33) It is therefore important to note that normal or low serum CK might not completely rule out active inflammatory myopathy MDA5-DM.

To our understanding, this is the first study that describes a direct comparison between MDA5-DM and ASyS patients in a southeast Asian population of mixed ethnicities. Given that IIM is rare and MDA5-DM RP-ILD appears to be reported more frequently in Asian cohorts, our findings add to the common knowledge of IIM, which remains rare. Our study has also adopted a comprehensive set of classification methodologies to classify the IIM patients, which would strengthen the comparative validity between our cohort and others. Our study is however limited by its single-centre retrospective design with small subgroup counts, dependence on line blot assay only without routine ELISA/immunoprecipitation confirmation of MSA/MAA presence, and potential unmeasured confounders (e.g., ferritin, KL-6, pulmonary function trajectories).

## Conclusion

In conclusion, this is the first study to our knowledge that describes a direct comparison between the clinical characteristics of MDA5-DM and ASyS in a southeast Asian population of mixed ethnicities. Our MDA5-DM and ASyS cohort has distinguished itself from the remaining IIM-subtypes with the higher preponderance for ILD, RP-ILD and arthritis, and a significantly lower prevalence of CAM. We have also described significant clinical characteristics in terms of DM-cutaneous features, higher risk ratio for RP-ILD and lower survival rate that would distinguish MDA5-DM from ASyS. Potential benefits can be derived from further studies to examine these IIM-subtypes that have a common predilection for lung involvement to facilitate more precise clinical diagnosis, prognostication and treatment.

## Data Availability

The datasets generated and/or analysed during the current study are not publicly available due to data protection policies of respective institutions and restrictions imposed by the ethics committee to ensure data privacy of patients. Aggregate data may be made available from the corresponding author on reasonable request.
